# TET1 knockdown inhibits the odontogenic differentiation potential of human dental pulp cells

**DOI:** 10.1038/ijos.2016.4

**Published:** 2016-05-27

**Authors:** Li-Jia Rao, Bai-Cheng Yi, Qi-Meng Li, Qiong Xu

**Affiliations:** 1 Guanghua School of Stomatology, Guangdong Provincial Key Laboratory of Stomatology, Sun Yat-sen University, Guangzhou, China; 2 Department of Stomatology, Nanshan Affiliated Hospital of Guangdong Medical College, Shenzhen, China

**Keywords:** DNA demethylation, human dental pulp cell, knockdown, odontogenic differentiation, ten–eleven translocation 1

## Abstract

Human dental pulp cells (hDPCs) possess the capacity to differentiate into odontoblast-like cells and generate reparative dentin in response to exogenous stimuli or injury. Ten–eleven translocation 1 (TET1) is a novel DNA methyldioxygenase that plays an important role in the promotion of DNA demethylation and transcriptional regulation in several cell lines. However, the role of TET1 in the biological functions of hDPCs is unknown. To investigate the effect of TET1 on the proliferation and odontogenic differentiation potential of hDPCs, a recombinant shRNA lentiviral vector was used to knock down TET1 expression in hDPCs. Following *TET1* knockdown, TET1 was significantly downregulated at both the mRNA and protein levels. Proliferation of the hDPCs was suppressed in the *TET1* knockdown groups. Alkaline phosphatase activity, the formation of mineralized nodules, and the expression levels of DSPP and DMP1 were all reduced in the *TET1*-knockdown hDPCs undergoing odontogenic differentiation. Based on these results, we concluded that *TET1* knockdown can prevent the proliferation and odontogenic differentiation of hDPCs, which suggests that TET1 may play an important role in dental pulp repair and regeneration.

## Introduction

Human dental pulp cells (hDPCs) are mesenchymal cells derived from the neural crest that exhibit plasticity and multipotency; these cells can generate reparative dentin to resist and repair injury due to infection or trauma.^1, 2^ A great deal of attention has been focused on the mechanisms involved in the odontogenic differentiation process for reparative dentine formation and dental pulp regeneration.^3, 4^ Numerous studies have proven that signal pathways play critical roles in regulating gene expression of the core transcriptional network of hDPCs, such as Wnt, transforming growth factor (TGF)-β, and Notch1 signaling.^5, 6, 7^ Several growth factors, including bone morphogenic proteins (BMPs) and growth and differentiation factor-5 (GDF-5), have been identified as chemotactic signals to recruit progenitor cells and stimulate their proliferation and differentiation in hDPCs.^8^ There is increasing evidence that epigenetic regulatory mechanisms such as histone acetylation and microRNAs are involved in reparative dentinogenesis.^9, 10, 11^ DNA methylation is a vital epigenetic modification that serves as a critical switch for gene expression, genomic consistency, and other processes of epigenetic inheritance.^12, 13, 14^ However, the role of DNA methylation in the regulation of the biological properties of hDPCs remains elusive.

Our previous study indicated that the DNA methyltransferase inhibitor 5-Aza-2'-deoxycytidine (5-Aza-CdR) promotes the odontogenic differentiation capacity of hDPCs, suggesting that DNA demethylation may provide a new mechanism for the regulation of odontogenic differentiation.^15^ Recent studies have shown that ten–eleven translocation 1 (TET1), a recently discovered DNA dioxygenase, could catalyze the addition of covalent hydroxyl modifications to methylated DNA and promote DNA demethylation. This reaction influences gene transcription, presumably by converting 5-methylcytosine (5mC) to 5-hydroxymethylcytosine (5hmC) at specific genes.^16, 17, 18, 19^ Increasing evidence indicates that TET1 is involved in the epigenetic regulation of proliferation and differentiation in various cells such as embryonic stem cells (ESCs), adult neural progenitor cells, muscle progenitor cells, and cancer cells.^20, 21, 22, 23, 24^ Previously, we reported that TET1 was expressed in hDPCs and that its expression increased during early natural differentiation and odontogenic induction.^25^ However, the role of TET1 in the odontogenic differentiation of hDPCs remains unknown.

In the present study, shRNA was used to knock down TET1 expression in hDPCs. The effect of TET1 on the proliferation and odontogenic differentiation of hDPCs was then investigated. Our results demonstrate the role of TET1-dependent DNA demethylation in the regulation of the differentiation potential of hDPCs.

## Materials and methods

### Cell culture

This study was approved by the Ethical Review Board of the Guanghua School of Stomatology of Sun Yat-sen University. All of the patients enrolled in this study gave written informed consent. hDPCs were isolated and cultivated as previously described by Gronthos *et al.*^26^ Briefly, pulp tissues were minced into small pieces and digested in a solution containing 3 mg·mL^−1^ collagenase type I (Gibco, Carlsbad, CA, USA) for 20 min at 37 °C. The pulp tissue was then cultured in complete medium containing Dulbecco minimum essential medium (DMEM) supplemented with 10% fetal bovine serum (FBS), 100 u·mL^−1^ penicillin, and 100 mg·mL^−1^ streptomycin (Gibco, Carlsbad, CA, USA) at 37 °C in an atmosphere of 95% O_2_ and 5% CO_2_. The media were changed every 3 days. When the cells reached 80% confluence, they were harvested using trypsin/ethylene diamine tetraacetic acid (EDTA) (Gibco, Carlsbad, CA, USA) and subcultured at a ratio of 1:3.

### Flow cytometry assay

The stem cell phenotypic markers of hDPCs were identified by flow cytometry; 10^5^ cells were resuspended in 100 μL phosphate-buffered saline (PBS) and incubated with primary STRO-1 and CD146 antibodies at 4 °C for 1 h, using 1:100 dilutions. The labeled cells were suspended in 100 μL PBS with 1 μL anti-mouse IgG conjugated with fluorescein isothiocyanate (Chemicon, Temecula, CA, USA) at 4 °C for 1 h and then examined with a FACS Calibur apparatus (Becton Dickinson, La Jolla, CA, USA).

### Knockdown of *TET1* in hDPCs using shRNA

To determine the role of TET1 in the differentiation of hDPCs, TET1 expression was knocked down using short hairpin RNA (shRNA). The TET1-targeting shRNA sequences (TET1 shRNA1: 5'-CAGAAGATTTAGAATTGAT-3' and TET1 shRNA-2: 5'-AGCTAATGAAGGTCCAGAAC-3') were designed and cloned into the hU6-MCS-CMV-Puromycin vector. The recombinant construct or a non-specific shRNA construct (control group), as well as three helper vectors (pLP1, pLP2, and pLP/VSVG), were transfected into 293FT cells. The viruses were collected 72 h later and transfected into hDPCs.

### Cell proliferation assay

The cell proliferation of hDPC was assessed using the Cell Counting Kit-8 (CCK8) assay (Dojindo, Kumamoto, Japan) according to the manufacturer's protocol. After 1, 2, 3, 4, 5, and 6 days of incubation, the supernatant of each group was removed, and the hDPCs were incubated in DMEM containing CCK8 for another 2 h at 37 °C. The optical density (OD) of each well was read at 450 nm using an automated microplate reader (Sunrise, Tecan, Switzerland).

### Odontogenic induction of hDPCs

The cells were seeded in six-well plates at a density of 1 × 10^5^ cells per well and cultured in DMEM containing 10% FBS until they reached 80% confluence. The medium was then replaced with odontogenic differentiation media containing DMEM supplemented with 5% FBS, 50 μg·mL^−1^ ascorbic acid, 10 mmol·L^−1^ b-glycerophosphate, and 10^−7^ mol·L^−1^ dexamethasone (Sigma-Aldrich, St Louis, MO, USA). The medium was changed every 3 days.

### Alkaline phosphatase activity analysis

After 7 days of incubation, the cells of each group were rinsed twice with ice-cold PBS (pH 7.4) and solubilized in 0.1% Triton X-100 for 15 h at 4 °C. An ALP assay kit (Nanjing Jiancheng Bioengineering Institute, Nanjing, China) was used to assess alkaline phosphatase (ALP) activity according to the manufacturer's instructions. The absorbance was measured at 520 nm using an automated microplate reader (Sunrise, Tecan, Switzerland). Total protein content was quantified using a bicinchoninic acid protein assay (Beyotime, Haimen, China) and the ALP levels were normalized to the total protein.

### Alizarin red S staining

The cells were seeded in six-well plates and mineralization was assessed *via* alizarin red staining after a culture period of 21 days. The mineralized nodules were observed and photographed using an inverted microscope (Zeiss, Jena, Germany). The amount of mineralization matrix was determined by dissolving alizarin red S in 100 g·L^−1^ cetylpyridinium chloride (CPC; Sigma-Aldrich, St Louis, MO, USA) and 10 mM sodium phosphate. The OD was read at 540 nm using an automated microplate reader (Sunrise, Tecan, Switzerland), and the quantitative measurements were made using the generated standard curve.

### Real-time quantitative polymerase chain reaction

Total RNA was extracted from the hDPCs using TRIzol reagent according to the manufacturer's instructions (Invitrogen, Carlsbad, CA, USA). Then, 2 μg of RNA was reverse-transcribed for cDNA synthesis using a RevertAid first strand cDNA synthesis kit (Fermentas, Ontario, Canada) and random primers. Real-time quantitative polymerase chain reaction (qRT-PCR) was performed using the LightCycler 480 SYBR Green I Master (Roche, Basel, Switzerland) with specific primers according to the manufacturer's instructions. The expression data were normalized to the geometric mean of the housekeeping gene GAPDH. The following primers were synthesized by Invitrogen (Life Technologies, Carlsbad, CA, USA): *TET1*: 5'-CATCAGTCAAGACTTTAAGCCCT-3'(forward), 5'-CGGGTGGTTTAGGTTCTGTTT-3'(reverse); *DSPP*: 5'-GCCACTTTCAGTCTTCAAAGAGA-3' (forward), 5'-GCCCAAATGCAAAAATATGTAA-3'(reverse); *DMP1*: 5'-AAAATTCTTTGTGAACTACGGAGG-3'(forward), 5'-GAGCACAGGATAATCCCCAA-3'(reverse); and *GAPDH*: 5'-GGCATGGACTGTGGTCATGAG-3'(forward), 5'-TGCACCACCAACTGCTTAGC-3' (reverse). The mRNA levels were normalized to the mRNA level of *GAPDH*.

### Western blotting analysis

The cells were harvested using RIPA lysis buffer (Beyotime, Haimen, China). In total, 40 μg of protein was subjected to 6% sodium dodecyl sulfate—polyacrylamide gel electrophoresis and transferred to polyvinylidene fluoride membranes (Millipore, Billerica, MA, USA) in transfer buffer containing 10% methanol. The membranes were blocked in TBST containing 5% skim milk at room temperature for 1 h and then incubated with an anti-TET1 (Genetex, Irvine, CA, USA), anti-DMP1 (Abcam, Cambridge, UK), anti-DSPP antibody (Abcam, Cambridge, UK), anti-β-ACTIN (Beyotime, Haimen, China) or an anti-GAPDH antibody (Beyotime, Haimen, China) overnight at 4 °C. After the cells were incubated with a secondary antibody (Abcam, Cambridge, UK) for 1 h at room temperature, the immunoreactive bands were developed using Amersham's enhanced chemiluminescence reagents (Millipore ECL Western Blotting Detection System, MA, USA) and observed using an ImageQuant LAS 4000 Mini system (GE Healthcare Life Sciences, Piscataway, NJ, USA). The blots were quantified and normalized using ImageJ 1.47 software (National Institutes of Health, Bethesda, MD, USA).

### Statistical analysis

Each experiment was performed in triplicate, and each set was repeated at least three times. The experimental groups were compared using one-way analysis of variance (ANOVA) or repeated-measures ANOVA with SPSS16.0 software (SPSS, Chicago, IL, USA). The values are expressed as the mean±standard deviation. All *P*-values are two-tailed, and *P*<0.05 was considered statistically significant.

## Results

### Identification of stem cell phenotypic markers in primary hDPCs

STRO-1^+^ and CD146^+^ have been shown to exhibit mesenchymal stem cell properties, and these markers have been used to identify dental pulp stem cells.^27^ STRO-1 and CD146 were identified using flow cytometry in primary hDPCs. The results revealed the expression of STRO-1 (23.68%) and CD146 (89.96%), indicating that hDPCs contain mesenchymal progenitors.

### TET1 expression levels in *TET1*-shRNA hDPCs

**Figure 1:**
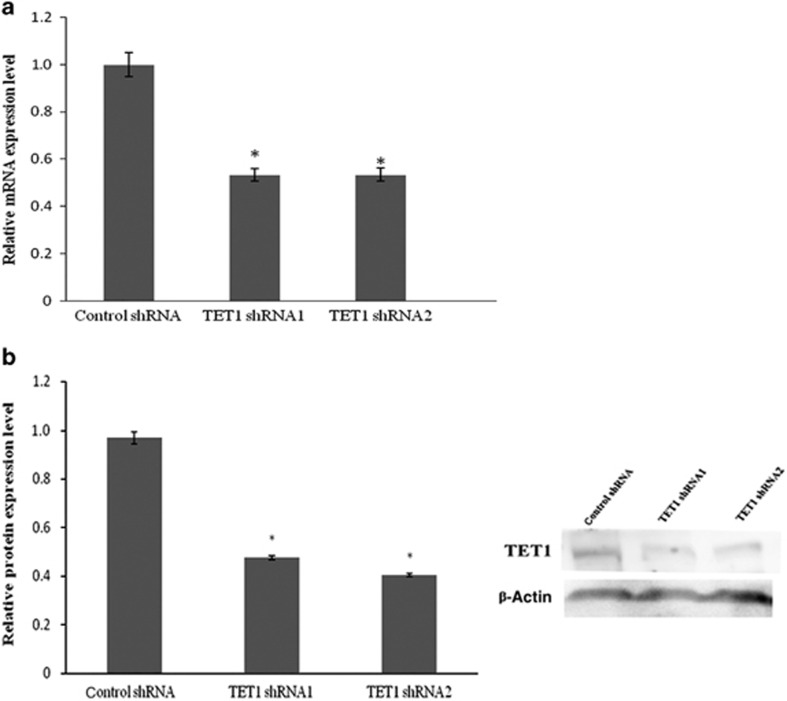
**The level of TET1 expression after *TET1* knockdown in hDPCs.** (**a**) TET1 mRNA expression was assessed using qRT-PCR. GAPDH was used as an internal control. (**b**) TET1 protein expression was assessed using western blotting and densitometric evaluation. β-ACTIN was used as an internal control. The expression level of TET1 decreased by approximately 50% in the shRNA1 and shRNA2 groups compared with the control group. All of the results represent the mean±standard deviation of three independent experiments (*n*=3). *Significant difference compared with the control (*P*<0.05). hDPC, human dental pulp cell; qRT-PCR, real-time quantitative polymerase chain reaction; shRNA, short hairpin RNA; TET1, ten–eleven translocation 1.

To investigate the role of TET1 in hDPCs, *TET1* was knocked down using shRNA. As shown in Figure 1a, the TET1 mRNA expression level decreased by approximately 50% in the shRNA1 and shRNA2 groups compared with the control group, which was consistent with the reduction in protein expression determined by western blotting (Figure 1b).

### Effect of *TET1* knockdown on the proliferation of hDPCs

**Figure 2:**
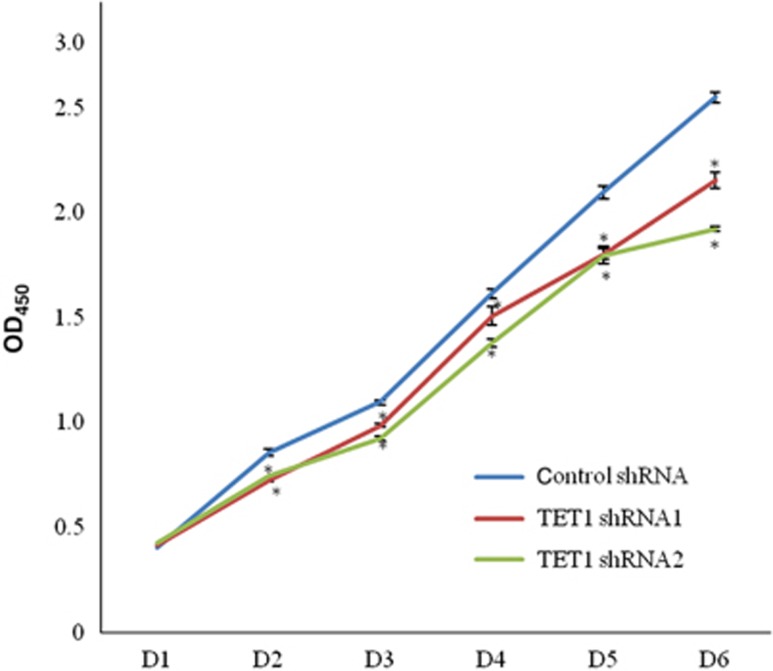
**Effect of *TET1* knockdown on the proliferation of hDPCs.** Cell growth of each group was measured using the CCK8 assay. The growth curves showed that the growth rates of the hDPC/shTET1 cells were significantly decreased from the second day onward. All of the results represent the mean±standard deviation of three independent experiments (*n*=3). *Significant difference compared with the control (*P*<0.05). D, day; hDPC, human dental pulp cell; TET 1, ten–eleven translocation 1.

To analyze the effect of *TET1* knockdown on the proliferation of the hDPCs, growth rates were measured using the CCK8 assay. As shown in Figure 2, the growth rates of the shRNA1 and shRNA2 groups decreased after 2, 3, 4, 5, and 6 days compared with the control group.

### Effects of *TET1* knockdown on the mineralization potential of hDPCs

**Figure 3:**
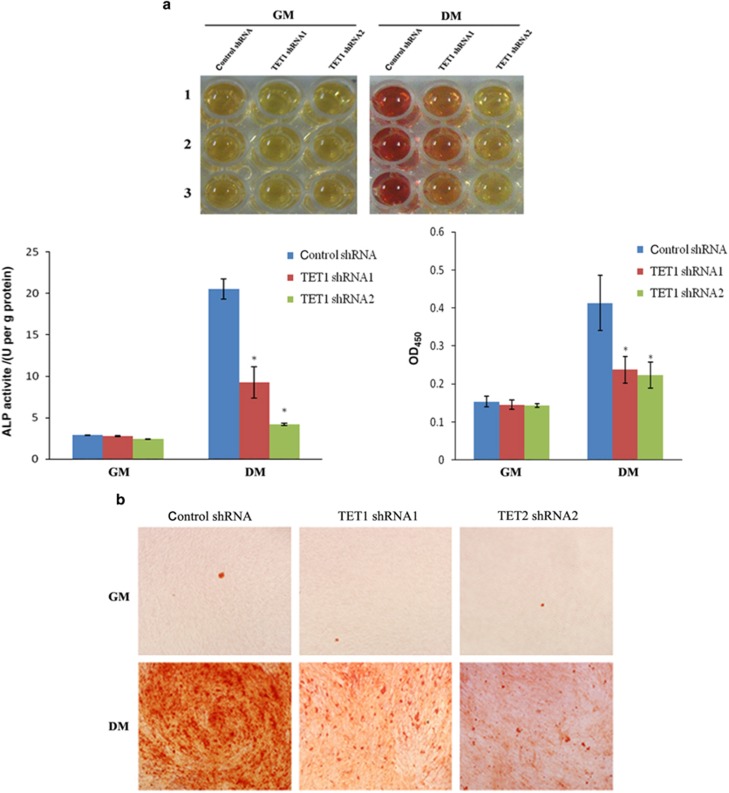
**Effect of *TET1* knockdown on the mineralization potential of hDPCs.** Cells were cultured in GM and DM. (**a**) ALP activity was measured at day 7. The ALP activity of all of the groups did not significantly differ under normal culture conditions. However, the ALP activity decreased by 54.92% in the shRNA1 group and by 79.52% in the shRNA2 group compared with the control group in the odontogenic differentiation medium. (**b**) Mineralization was analyzed using alizarin red S staining at day 21. The formation of mineralized nodules was inhibited in the *TET1*-knockdown hDPCs undergoing odontogenic induction. All of the results represent the mean±standard deviation of three independent experiments (*n*=3). *Significant difference compared with the control (*P*<0.05). ALP, alkaline phosphatase; DM, differentiation medium; GM, growth medium; hDPC, human dental pulp cell; shRNA, short hairpin RNA; TET1, ten–eleven translocation 1.

To determine the effect of *TET1* knockdown on the mineralization potential of hDPCs, ALP activity and the formation of mineralized nodules were assessed. The ALP activity of all of the groups did not significantly differ under normal culture conditions. However, in the odontogenic induction medium, ALP activity decreased by 54.92% in the shRNA1 group and by 79.52% in the shRNA2 group compared with the control group (Figure 3a). Similarly, alizarin red S staining indicated that the formation of mineralized nodules was inhibited in the *TET1*-knockdown hDPCs undergoing odontogenic induction (Figure 3b).

### Effects of *TET1* knockdown on odontogenic differentiation markers in hDPCs

**Figure 4:**
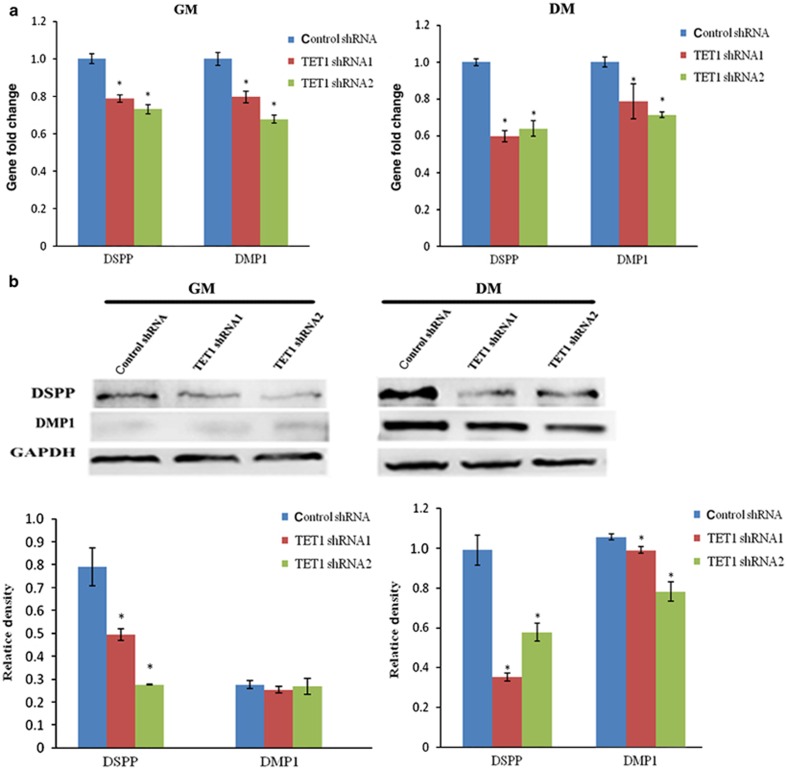
**Effect of *TET1* knockdown on DSPP and DMP1 in hDPCs.** (**a**) The mRNA expression levels of DSPP and DMP1 were determined using qRT-PCR. GAPDH was used as an internal control. The mRNA expression levels of DMP1 and DSPP decreased in the *TET1*-shRNA groups with or without odontogenic induction. (**b**) Protein expression levels of DMP1 and DSPP were assessed by western blot. GAPDH was used as an internal control. The protein expression of DSPP decreased in both culture media, whereas the protein expression of DMP1 did not significantly differ under normal culture conditions but decreased in the *TET1*-knockdown hDPCs undergoing odontogenic induction. All of the results represent the mean±standard deviation of three independent experiments (*n*=3). *Significant difference compared with the control (*P*<0.05). DM, differentiation medium; GM, growth medium; hDPC, human dental pulp cell; qRT-PCR, real-time quantitative polymerase chain reaction; shRNA, short hairpin RNA; TET1, ten–eleven translocation 1.

To further identify the role of TET1 in the regulation of the odontogenic differentiation potential of hDPCs, expression of the odontogenic markers DSPP and DMP1 was detected using qRT-PCR and western blotting. *TET1*-shRNA led to a decrease in the mRNA expression levels of DMP1 and DSPP with or without odontogenic induction (Figure 4a). The protein expression level of DSPP decreased in culture media, whereas the protein expression of DMP1 did not significantly differ in normal culture conditions but decreased in the *TET1*-shRNA groups after odontogenic induction (Figure 4b).

## Discussion

Elucidating the molecular mechanisms that regulate the odontogenic differentiation of hDPCs would contribute greatly to advancing treatment strategies for regenerative endodontics. Our recent studies indicated that DNA demethylation may provide a way to creatively illustrate this problem.^15, 25^ TET1 is a key protein in the DNA demethylation pathway and has a profound impact on cell self-renewal and on the determination of lineage.^28, 29^ However, the mechanism by which TET1 is involved in the differentiation program of hDPCs remains unclear.

TET1 is known to catalyze the conversion of 5mC to 5hmC.^16^ 5hmC can be further oxidized to 5-formylcytosine and 5-carboxylcytosine. These derivatives, the production of which is mediated by TET1, can be actively removed from the genome by thymine-DNA glycosylase through a base excision repair pathway and can then convert back to 5mC.^30, 31^ This process is called active DNA demethylation, which is associated with the activation of genes.^32^ Recent studies have demonstrated that TET1 is involved in the control of active and passive demethylation via different mechanisms and thus plays a vital role in the modulation of primordial germ cell formation, embryonic development, stem cell pluripotency, and nerve and brain development.^20, 33, 34, 35^ Our previous study demonstrated that TET1 is present in the hDPCs and that its expression increases in a time-dependent manner during odontogenic induction.^25^ The present study further investigated the role of TET1 in the proliferation and odontogenic differentiation of hDPCs.

Zhang *et al.*^21^ observed that the loss of *Tet1* downregulated a cohort of genes involved in the proliferation of adult neural progenitor cells and impaired hippocampal neurogenesis in mice, which was accompanied by poor learning and memory. *Tet1* depletion inhibits the growth of NIH3T3 cells by blocking cyclin D1 accumulation in G1 phase, inhibiting Rb phosphorylation and consequently delaying entrance to G1/S phase.^36^ The reduction of *TET1* significantly downregulated proliferating cell nuclear antigen, thus inhibiting cell proliferation in human uterine leiomyoma.^37^ In the present study, *TET1* knockdown suppressed cell growth from the second day onward, suggesting that the loss of TET1 inhibited the proliferation of the hDPCs. The inhibitory effect of *TET1*-shRNA on the proliferation of hDPCs was in accordance with these previous reports.

TET1 can influence the balance between 5hmC and 5mC in the genome, which is inextricably associated with lineage commitment.^13, 19^ Wu *et al.*^28^ found that Tet1 has a dual function in transcriptional regulation in mouse ESCs. It binds to and affects both repressed and actively transcribed genes. TET1-mediated hypomethylation of promoter regions leads to the upregulation of genes related to pluripotency and ES cell maintenance. Meanwhile, Tet1 also contributes to gene silencing by facilitating the recruitment of polycomb repressive complex 2 or SIN3A to genes that function in development and differentiation.^38^ Furthermore, TET1 could maintain a hypomethylated state at the regions to which it was bound, but its depletion did not lead to downregulation of all the TET1 targets.^28^ With regard to somatic cells, Fujiki *et al.*^39^ found that TET1 catalyzes the conversion of 5mC to 5hmC by binding to the PPARgamma co-activator complex, thereby inducing region-specific demethylation and activating the adipocyte differentiation process. Jin *et al.*^40^ recently reported that TET1 does not purposely decrease methylation levels; instead, it specifically prevents the spread of aberrant methylation into CpG islands in differentiated cells (HEK293T cells). These new findings suggest that TET1-dependent demethylation plays an important role in the lineage determination, but the underlying mechanism is complex and remains controversial. In the present study, *TET1* knockdown decreased ALP activity and the formation of mineralized nodules, and downregulated the expression levels of DSPP and DMP1, which indicates that TET1 is positively involved in the regulation of the odontogenic differentiation of hDPCs. The exact mechanisms by which the TET1 enzyme regulates the differentiation properties of hDPCs are currently being investigated in our laboratory.

In conclusion, this study demonstrated that *TET1* knockdown can suppress cell growth and prevent the odontogenic differentiation of hDPCs by inhibiting ALP activity, mineralized nodule formation, and the expression of DSPP and DMP1. These findings indicate that TET1 may promote the proliferation and odontogenic differentiation of hDPCs. More studies are necessary to further elucidate the epigenetic mechanisms by which TET1-dependent demethylation regulates the biological characteristics of hDPCs.
